# A novel biosurfactant producing *Kocuria rosea* ABR6 as potential strain in oil sludge recovery and lubrication

**DOI:** 10.1186/s13568-021-01283-9

**Published:** 2021-09-22

**Authors:** Elham Akbari, Behnam Rasekh, Keivan Beheshti Maal, Farahnaz Karbasiun, Fatemeh Yazdian, Zarrindokht Emami-Karvani, Reza Peighami

**Affiliations:** 1 Department of Microbiology, Faculty of Biological Sciences, Falavarjan Branch, Islamic Azad University, Isfahan, Iran; 2 Environment and Biotechnology Research Division, Research Institute of Petroleum Industry (RIPI), P.O. Box: 14665-137, Tehran, Iran; 3 Department of Microbiology, Faculty of Microbiology Sciences, North Tehran Branch, Islamic Azad University, Tehran, Iran; 4 Faculty of New Science and Technology, University of Tehran, Tehran, Iran; 5 Biotechnology Group, Department of Chemical Engineering, Tarbiat Modares University, Tehran, Iran

**Keywords:** Biosurfactant, *Kocuria rosea*, Lubrication, Oil Sludge, Response Surface Methodology

## Abstract

Biosurfactants are amphiphilic molecules composed of a hydrophilic and hydrophobic moiety and had the ability to penetrate into different phases to reduce the surface tension. This features caused to oil recovery, lubrication and facilities of crude oil in pipeline. In current research Biosurfactant-producing strain was isolated from the storage tanks of the Isfahan Oil Refining Company in Iran, and screened by oil expansion test, droplet collapse, and surface tension reduction measurement. Hydrocarbon recovery from crude oil sludge was measured under constant conditions. The effect of factoring biosource lubrication on crude oil in pipelines was investigated in vitro. Also, the optimization of biosurfactant production in different conditions was measured as a single factor and using Response Surface Method (RSM). The best biosurfactant-producing bacterium was identified as *Kocuria rosea* ABR6, and its sequence was registered in the gene bank with access number of MK100469. Chemical analysis proved that the produced biosurfactant was a lipopeptide. 7% of crude oil was recovered from petroleum sludge by biosurfactant obtained from *Kocuria rosea* ABR6. Also, the speed of crude oil transfer in pipelines was upgraded as it could be said that for a certain distance the transfer time reduced from 64 to 35 s. The highest biosurfactant production was measured at pH 9, aeration rate of 120 rpm and 96 h after incubation. The use of biosurfactants produced by *Kocuria rosea* ABR6 is recommended to remove oil sludge and lubricate oil in pipelines recommended in the oil industry.

## Key points

- To improve recovery and prevent waste accumulation in crude oil storage tank, application of biosurfactant suggested.
- The isolated bacteria have high lubrication capacity.
- One of the significant problems in petroleum industry is biodegradability of Oil derivatives like oil sludge.

## Introduction

In recent years, the control and prevention of chemical pollutants in the petroleum industry always have been a worldwide issue (Varjani and Upasani 2017). There are three physical, chemical, and biological methods for treating these pollutants. However, these mechanisms have numerous disadvantages e.g. in most cases the final product is usually toxic. In addition, there is an urgent need for sustainable cognition and eco-friendly methods which require a lesser amount of chemicals, are economically viable, and produce non toxic natural final products. One of the chemical pollutants in the petroleum industry is emulsion of different petroleum hydrocarbons, solid particles, heavy metals, and highly toxic water, named oil sludge. Removal of these components requires expensive and time-consuming processes. Due to its hazardous nature and increased quantities around the world, it's necessary to clarify this problem (Lima et al. 2011). Expulsion of oily sludge from storage tanks can be performed by using biosurfactants to decline viscosity and recovery and refinery of oil (Banat et al. 1991). Other treatments of oily sludge such as composting and land farming may have considerable applicability and a low operating budget for large-scale treatment, but their microbial degradation process is time-consuming. Selection of proper method depends on sludge features, disposal regulatory requirements, costs, time constraints (Hu et al. 2013). The use of Biosurfactants in the industry is the most important achievement of the twenty-first century (Singh et al. 2018). Biosurfactants are amphiphilic surface-active agents secreted from microorganisms. Bioactive molecules which are able to decrease the surface tension between an aqueous mixture and hydrocarbon (Shekhar et al. 2015). In recent years, the application of biosurfactants has increased significantly, due to the non-toxic, inexpensive economic value, biodegradable nature. Bioremediation process is based on an integrated approach employing microbial communities such as fungi, actinomycetes, bacteria, and earthworms (Suganthi et al. 2018). On the other hand, the global market for surfactant is amazing and, the market is also, expected to grow to USD 39.86 Billion by 2021 (Markets and Markets 2017; Hart 2014). It is considered as a viable process for administration of organic pollutants-rich solid wastes and wastewater. Many studies have suggested using bacteria as a biosurfactant to recovery and removing oily sludge. Many studies have used bacteria as biosurfactant to recovery oily sludge, *Pseudomonas aeruginosa F-2* (Yan et al. 2012) from refinery oily sludge, *Bacillus subtilis 3KP* (Ni’matuzahroh et al. 2016) from the petroleum sludge, *Pseudomonas balearica strain Z8* (Nejad et al. 2020) from oily sludge wastes. According to many studies, Oily sludge is one of the most important solid wastes generated in the petroleum industry, and understanding the mechanism of degradation it would be significant, so we decided to investigate biosurfactants as the best choice (Hu et al. 2013). The utilize of environmental nanotechnologies (E_nano) is a complex process to resolve the problems as best as associated with the petroleum industry, (Younis et al. 2020) we carried out ZnO as E-Nano in our project. There aren’t many documents to investigate about biosurfactant production by *Kocuria rosea*. The main aims of this study is introduce suitable native biosurfactant producing bacteria from petroleum reservoir tanks and improve biosurfactant production by optimization. Then lubrication of crude oil in pipeline model and oil recovery from oily sludge by this biosurfactant have been evaluated.

## Material and methods

### Sample collection

The sample, collected from crude oil storage tanks located in Isfahan Oil Refining Company, Isfahan, Iran. Afterwards, transferred to the laboratory under controlled conditions.

### Isolation of biosurfactant producing bacteria

In order to isolate the bacteria, Bushnell Hass culture medium was used. Culture medium contains (g L^−1^): KH2PO4, 1; 0.2; K2HPO1; NH4NO3, MgSO4. 7H2O 1; FeCl3, 0.002; CaCl2, 0.02; and crude oil, 1% (v/v), and the pH was adjusted to 7 using NaOH 1 M. The culture medium was sterilized by autoclave; 10 ml of each sample were inoculated in a 500 ml medium and incubated for 10 days at 30 C and 100 rpm. Then 1 ml of inoculum was added to the culture medium (1.8 g L^−1^ agar). In the next step, bacteria which grown on the specific medium were transferred to an olive broth with the following compounds (g L^−1^): MgSO4. 7H2O, 0.2; K2HPO4, 1; NH4NO3, 1; FeCl3, 0.002; CaCl2, 0.02; yeast extract and olive oil, 1% (v/v). Olive oil was added, and the pH was adjusted to 7. After autoclaving, 1% of pre culture was shifted to the olive broth and incubated for 4 days at 30° C and 100 rpm (Cameotra and Singh 2008; EI-Sheshtawy and Doheim 2014).

### Identification of bacterial isolate

To better and certain identification, based on the other studies, positive or negative to gram stain test and some routine biochemistry tests (Jessim et al. 2020), like urease, oxidase, utilization of inulin, citrate utilization test, phosphatase tests, gelatinase, nitrate reduction tests arabinose, and *N*-acetyl-l-glutamic acid amylase (Kandi et al. 2016).

To molecular test, Bacterial genomic DNA was extracted from the bacterial isolate grown on nutrient broth using standard protocol. PCR was carried out as formerly described (Akbari et al. 2016). 16S rDNA amplicon from isolated strain was sequenced, and the information was searched using NCBI-BLAST search tool for identification of the strain type. The sequence was submitted to the NCBI Gene Bank (Akbari et al. 2018).

### Evaluation of biosurfactant production by isolated bacteria

Possibility of biosurfactant presence was investigated using oil displacement method. In short, 50 μl of crude oil was mixed to a 10 cm diameter plate containing 10 ml of distilled water. The cell free culture broth (15 μl) was then added to the oil surface. The plates with clear zone were scored as positive indicative of the present of biosurfactant production (Mousavi et al. 2015; Nejad et al. 2020; Liu et al. 2020). Surface tension of supernatant was measured by tensiometer (KRÜSS KIOT K9, Switzerland) and reported as mN/m. The surface tension of the water was considered as a control. Also extraction of biosurfactant from isolated bacteria was performed according to the technique mentioned by Jorfi et al. (2013). After centrifugation at 10,000*g* for 15 min in order to eliminate the bacterial cells, and the pH was adjusted by adding 2.0 ml of HCL to the biosurfactant. The precipitate was separated by centrifugation (10,000*g* for 20 min) and then extracted with chloroform: methanol (2:1, v/v) mixture. In order to have crude biosurfactant, solvent evaporation under vacuum was used.

### Treatment of oily sludge with biosurfactant

To preparate the crude oil from oil sludge in laboratory conditions, the present process was performed according to the method introduced by Lima et al. 40 g of petroleum sludge obtained from the bottom of crude oil storage tanks autoclaved from Khark refinery. Then 50 ml of sterilized distilled water was added to it. Then 10 ml of the medium culture of biosurfactant were inoculated after 96 h of incubation. Positive control was prepared by adding 10 ml of Twin 80 and negative control was prepared without adding chemical surfactant and biosurfactant. After 5 days Incubation at ambient temperature and aeration at 100 rpm, oily sludge was emulsified. Then emulsion by adding 1 ml of magnesium nitrate solution was broken, the aqueous phase was separated and the amount of recycled oil was measured (Lima et al. 2011; Cameotra and Singh 2008; Akbari et al. 2020).

### Crude oil lubrication by biosurfactant

In present study, the isolated strain was cultured in Bushnell Hass medium at 30 °C and 100 rpm. After 96 h incubation, in order to ensure from biosurfactant production, emulsification index, and oil displacement method were developed. The pipeline was designed under laboratory condition. Gross biosurfactant was poured in the crude oil from the collected sample in a ratio of one-fifth as the optimal ratio and it was performed under mixing with a specified speed, and the system output was then passed through a 40 cm duct at a 65° angle. Duct passage time was recorded before and after mixing (Amani and Kariminezhad 2016).

### Chemical analysis TLC and FTIR

#### Product analysis by thin-layer chromatography (TLC)

In order to chromatograph, a thin layer of silica 60 paper with dimensions of 15.5 cm was arranged. 0.1 mg of dried biosurfactant dissolved in 10 μl of 90% ethanol and 5 µl of sample was dotted at a distance of 1 cm from the edge of the paper. The solvent system that was used as the mobile phase including chloroform, methanol, acetic acid with a ratio of 65/15/2 v/v/v was selected. For staining solution containing 15 Ursinol and 8.2 ml of 60% sulfuric acid in 42 ml of distilled water sprayed on paper and dried at 100 °C for 10 min and the spots were examined (Camacho-Chab et al. 2013).

#### Product analysis by Fourier-transform infrared spectroscopy (FTIR)

FTIR (BRUKER,) was performed to show the presence of chemical functional groups in bio surfactant produced by isolated bacteria. Lyophilize was performed to extracted biosurfactant and biosurfactant analysis was performed in assay range of 400–4000 cm^−1^ (Yaraguppi et al. 2020).

### Biodegradation analysis of crude oil

BH media selected to evaluate the ability of bacteria isolated from crude oil (100 ml) supplemented with 1% crude oil. Upon incubation at pH 7 and 30 °C, 160 rpm for 72 h, the grown cultures were centrifuged at 100 rpm, 4 °C for 10 min to pellet down the cells. For Isolated bacteria, crude oil was used as carbon source. This was investigated by the weighing the residual crude oil based on a gravimetric method wherein the residual crude oil in the cell-free supernatant was extracted in an equal volume of n-hexane and separated using a rotary vacuum evaporator (Sharma et al. 2019).

### Optimization of culture conditions for biosurfactant production

In order to determine biosurfactant production ability of the isolated bacteria, we decided to carry out single factor optimization and multi-factor optimization by Response Surfaces methodology (RSM). In the first section, we overviewed 40 experiments, and according to the results, we detected 15 experiments, which showed in Table 1. In this study, for performing single factor optimization for biosurfactant production, the five factors including pH, temperature, carbon and nitrogen sources, agitation and time condition were considered. The culture conditions were based on pH (7, 8, 9, and 10), temperature1 and carbon source (oil olive, glucose, tribotirin, crude oil) and nitrogen source (peptone, NH4NO3, NaNO3, triptone, yeast extract), the biosurfactant production was also evaluated under agitation (80, 100, 120, 140 rpm) and time conditions. During the experiments, when each factor was examined, the other four were maintained as constant. To determine the importance of the factors and to better understand interactions between them analysis using ANOVA table was developed (Liu et al. 2020).

**Table 1:** Experiments of second step designed by response surface analysis

Experiment	Factor number1 Agitation rate	Factor number2 Incubation	Factor number3 pH
1	140	120	9
2	140	72	8
3	140	96	10
4	100	120	9
5	100	96	10
6	100	72	8
7	120	96	9
8	120	96	8
9	120	120	10
10	120	72	10
11	120	96	9
12	120	96	8
13	120	72	9
14	120	96	9
15	120	120	10

## Result

**Fig. 1:**
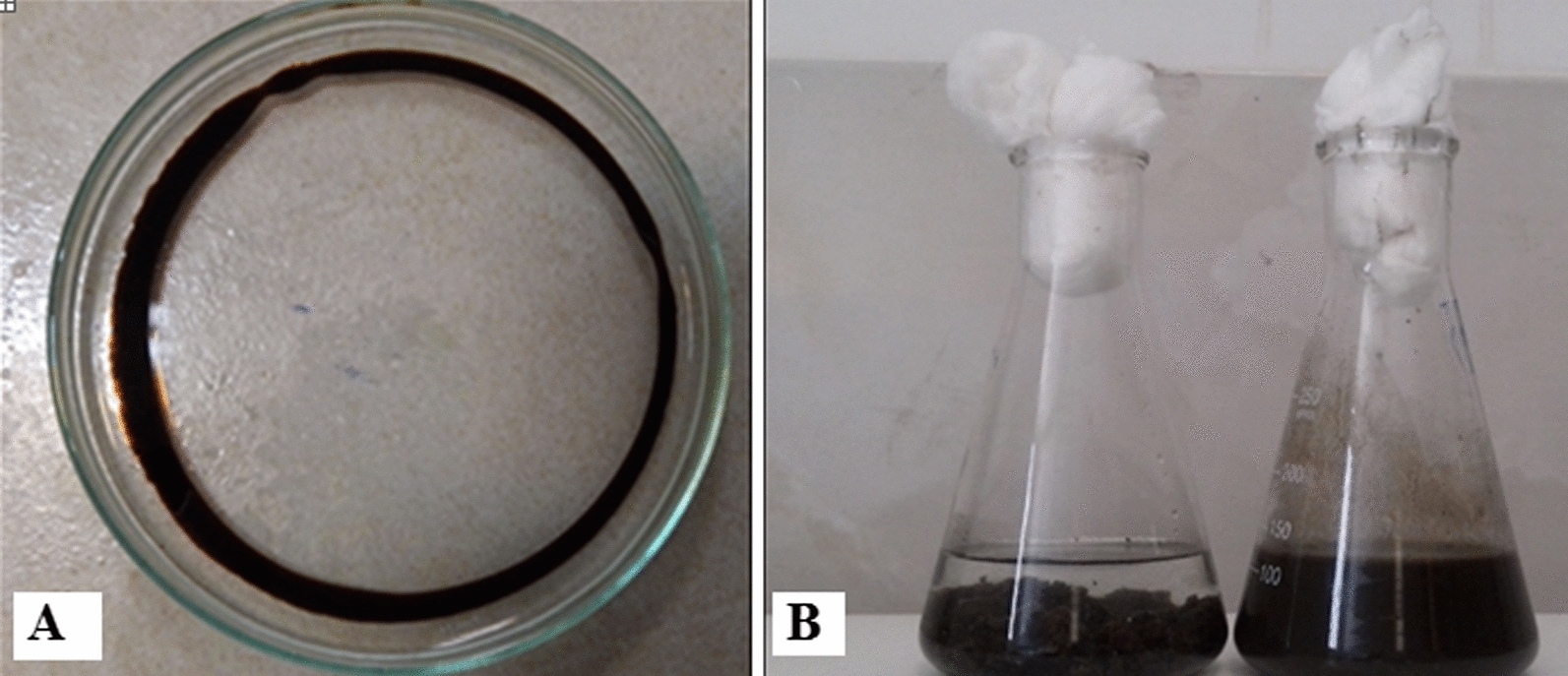
**A** Oil displacement test by supernatant obtained from *Kocuria rosea* ABR6. **B** The emulsion stability of oily sludge obtained from culture medium of *Kocuria rosea* ABR6 after 7 days' incubation

In current research, several bacteria strains were isolated. Because the dominant strain in enrichment culture (Bushnell Hass medium) was W11 isolate, bacterial production was examined. The screening revealed that W11 produced a considerable amount of biosurfactant, and reduced the surface tension from 72 mN/m to 31.6 mN/m. Also; the diameter of the oil expansion halo was measured at 9 cm (Fig. 1A). Drop collapse in less than a minute pointed up the presence of biosurfactant in the Bushnell Hass medium. The drop collapse test to survey biosurfactant Produced by W11 was positive. Isolated bacteria were measured by standard screening technique like, oil displacement method, oil drop collapse method (DCM), surface tension (SFT) measurement and emulsification index (Soltanighias et al. 2019). This proved its ability to produce biosurfactants and selected for further oil recovery analysis, optimization, and Biodegradation analysis.

### Isolation of Biosurfactant Producing Bacteria

The isolated bacteria showed a positive growth in the selective culture medium (olive broth and Buschnel Hass) and this strain we named, W11. To certain identification, biochemistry tests were helpful, e.g. PCR of purified bacterial DNA with universal primers presented the 1500 bp band in gel electrophoresis. The analysis of the genomic sequence of 16S rDNA using Finch TV and thence BLASTN approved that W11 from oil sludge sample has been associated with *Kocuria rosea* and was named *Kocuria rosea* ABR6.The genomic sequence of 16S rDNA was deposited in the NCBI under the accession number of MK100469*.* Also this strain deposited in Petroleum Biotechnology Culture Collection as *kocuria rosea* PBCC1167.

### Treatment of oily sludge with biosurfactant

The medium culture of *Kocuria rosea ABR6* after 72 h incubation with 100 rpm at 30 °C was performed to decrease the oily sludge with sharp viscosity in crude oil storage tank. As a result, the treatment of petroleum sludge with biosurfactant from the isolated *Kocuria rosea ABR6*, 50% of crude oil was recycled in laboratory conditions (Fig. 1B). In the positive control sample, 75% of crude oil was recovered from petroleum sludge, provided, in the negative control sample, only 3% of the crude oil is recycled.

### Crude oil lubrication by biosurfactant

For a better describing the effect of Crude oil lubrication using Biosurfactant, we designed an experiment on crude oil in pipelines in vitro. The Biosurfactant produced by isolated bacteria accelerated the movement of crude oil, as the crude oil movement time decreased from 66 to 39 s.

### Chemical analysis TLC and FTIR

**Fig. 2:**
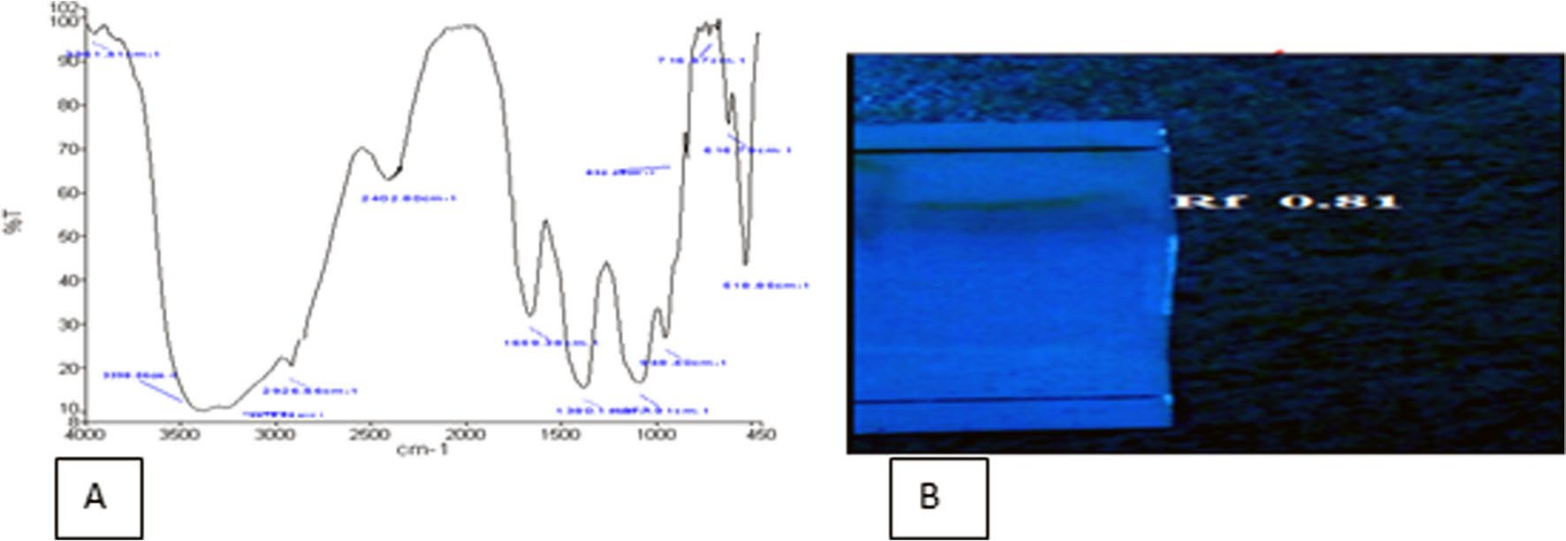
**A** Characterization of biosurfactan using FTIR analysis from *Kocuria rosea* ABR6. **B** Chromatogram biosurfactant produced by *Kocuria rosea* ABR6

Thin-layer chromatography (TLC) was used to separate non-volatile mixtures. After accomplishment of 72 h, the rest of oil and biosurfactant in the supernatant was controlled with chromatographic technique. This study was performed with abiotic sample introduced as a control. As illustrated in Fig. 2B, Rf 0.81 was observed.

FTIR analysis was performed to characterize the biosurfactant type secreted from *Kocuria rosea ABR6*. According to spectrum FTIR (Fig. 2A), A stretch around 1380 cm^−1^ corresponds to the presence of –CH_3_ and –CH_2_ groups in aliphatic chains of lipids. A broad band at 2926 cm^−1^ represents the O–H stretching vibrations from free hydroxyl groups. Regions around 2926 cm^−1^ signify alcohols and phenols. Peak in the region of 518 cm^−1^ may be likely due to the presence of disulfides in the molecule. Peak around 2402 cm^−1^ may be representing the P–H in the phosphine. Peak around 948 cm^−1^ may be due to occurrence of P–O–R stretch of ester group. Peak around 3398 cm^−1^ reveals the presence of RCONH2 related to amino acids. Peak near 1659 cm^−1^ indicate to the C=C from alkene of bacteria protein, also, peak near 832 cm^−1^, 616 cm^−1^ and 716 cm^−1^ due to the presence of alkene. Peak around 936 cm^−1^ may be attributed to OH, Carboxylic group. The FTIR analysis demonstrated the biosurfactant produced by the *Kocuria rosea ABR6* was the lipopeptide.

### Biodegradation analysis of crude oil

Following the research, we found out the biosurfactant production utilizes various carbons as energy source. In this study, the ability of *Kocuria rosea ABR6* in utilizing crude oil as a carbon source and producing biosurfactant was explored. The percentage of crude oil biodegradation by biosurfactant sounds fast. This may be because that the microorganisms in the oil sludge have the ability of using the remaining crude oil as a source of carbon and energy. In sum, degradation of crude oil was reached as 22%.

### Optimization of bacterial growth in order to maximize biosurfactant production

#### Single factor test3

**Fig. 3:**
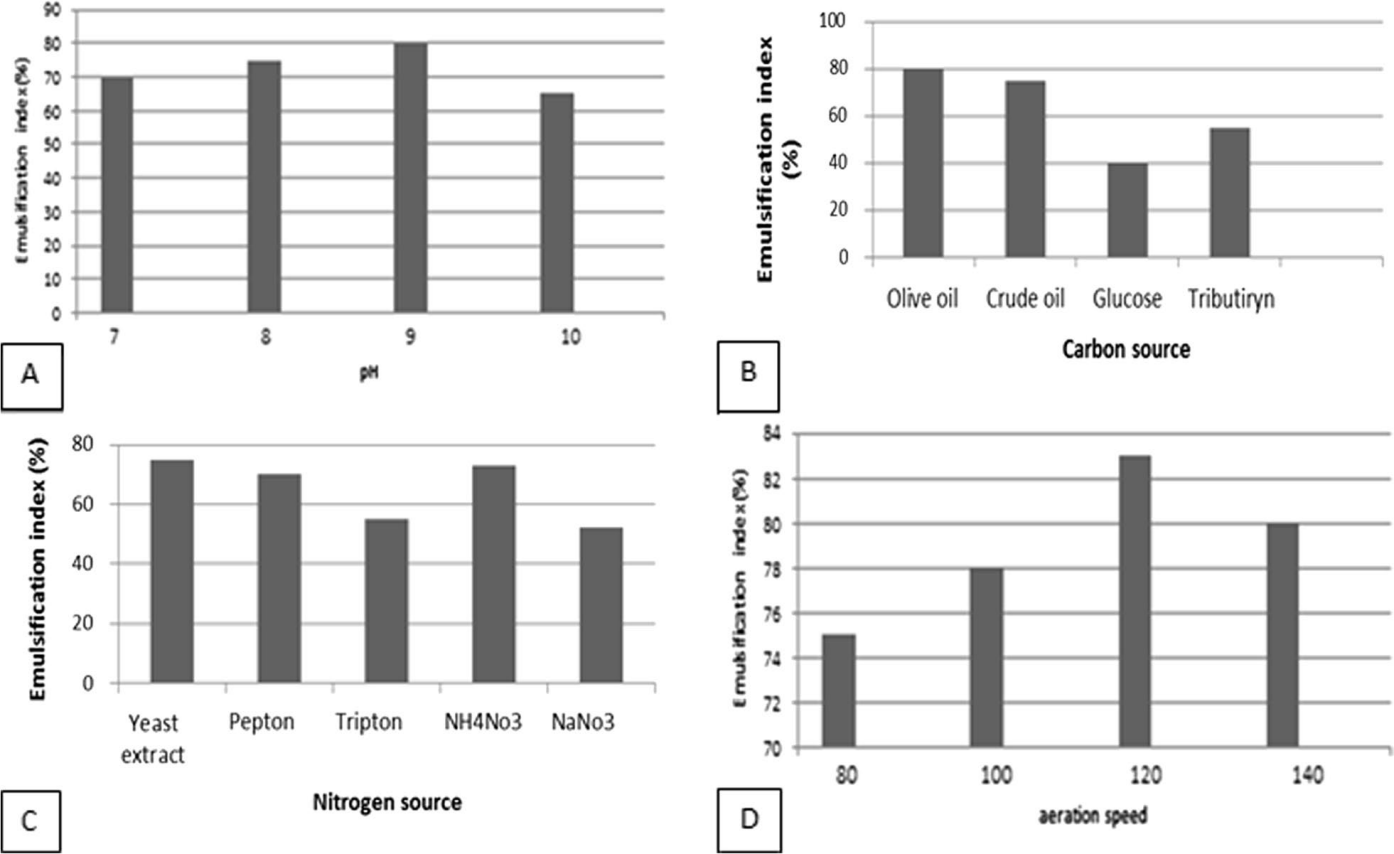
**A** Impact of pH in biosurfactant produced by *Kocuria rosea* ABR6, **B** impact of carbon sources biosurfactant produced by *Kocuria rosea* ABR6. **C** Impact of nitrogen sources biosurfactant produced by *Kocuria rosea* ABR6. **D** Impact of agitation biosurfactant produced by *Kocuria rosea* ABR6

Impact of pH: To increase the amount of biosurfactant production by the *Kocuria rosea ABR6* during the selective condition, five parameters need to be optimized. According to the results, the highest emulsification was related to pH 9 and around 80% (Fig. 3A) respectively, emulsification of the isolated bacteria in pH 7, 8, 9, and 10 were 70%, 75%, 80%, and 65%. Impact of carbon sources: The most production of biosurfactant resulted to be 80% and was related to olive oil about 80%, also fermentation of glucose, tributyrin, crude oil by isolated strain were 40%, 53%, 75% (Fig. 3B). Impact of nitrogen sources: In order to find the best nitrogen sources, numbers indicated yeast extract with 75% was at the best nitrogen source. Other sources include, peptone, NH4NO3, NaNO3, and tryptone were 70%, 73%, 52%, and 55%. (Fig. 3C). Impact of agitation: results show the best agitation was 120 rpm, the rest of the results of agitation under 80 rpm, 100 rpm, 120 rpm, and 140 rpm were, 75%, 78%, 83%, and 80% (Fig. 3D).

### Response surface analysis

**Fig. 4:**
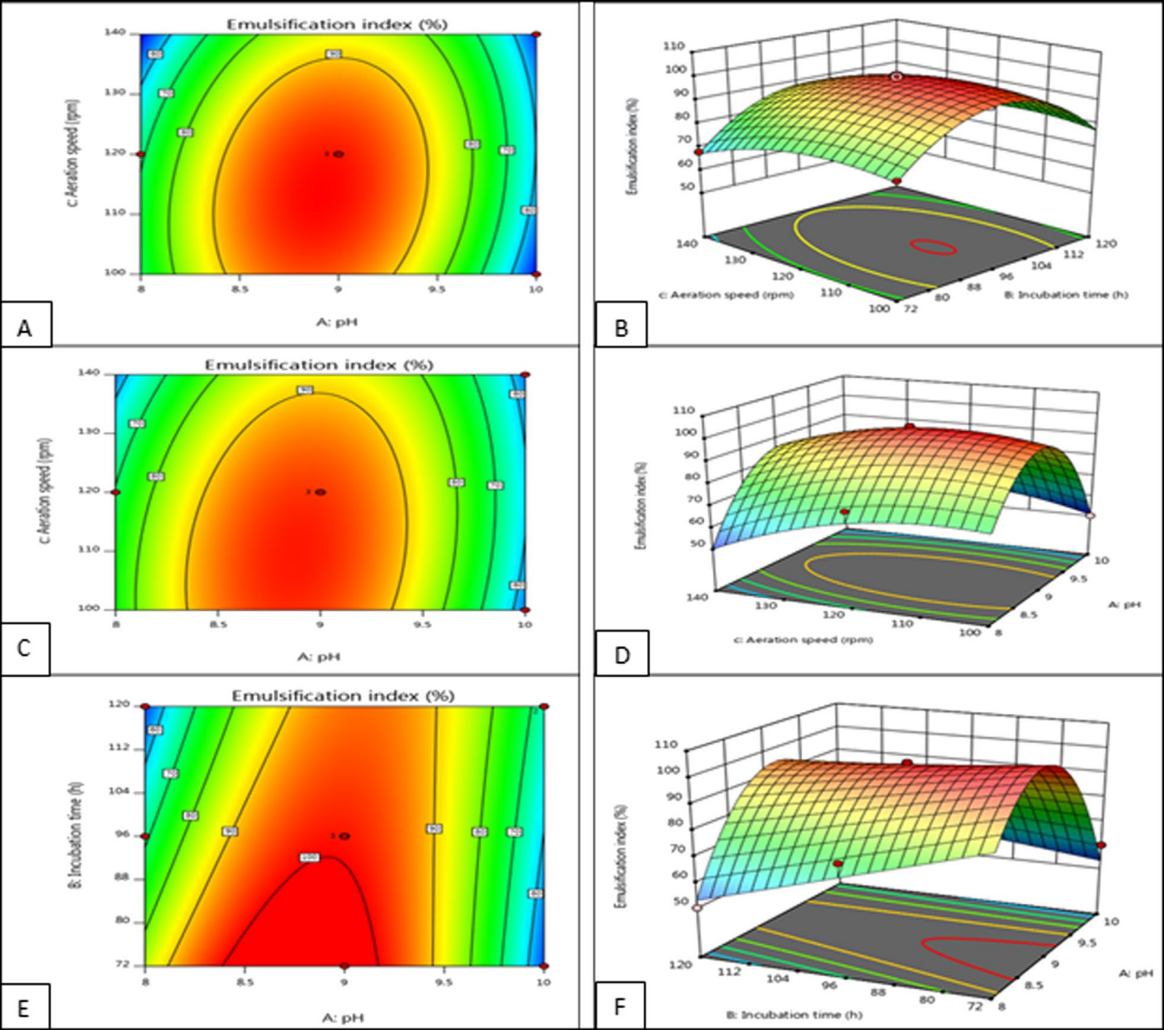
**A** Contour curve the effect of aeration speed factors and incubation time *kocuria rosea* ABR6, **B** three-dimensional (3D) curve of the effect of aeration velocity factors and incubation time, **C** contour curve the effect of pH and aeration rate factors *kocuria rosea* ABR6, **D** three-dimensional (3D) curve of the effect of pH and aeration factors *kocuria rosea* ABR6, **E** contour curve the effect of pH factors and incubation time *kocuria rosea* ABR6, **F** three-dimensional (3D) curve of the influence of pH factors and incubation time

The response surface analysis results are showed in Fig. 4. The highest growth was related to 72 h after incubation; hence, the highest emulsification was around 96 percentages. According to graph after 96 h, isolated strain showed the most biosurfactant production. It shows, *Kocuria rosea* had maximum biosurfactant production in stationary phase. According to Fig. 4, pH 9 provided the best condition for biosurfactant production in this case study. Based on the emulsification activity around 97.38, aeration speed optimally reported to be 120 rpm. In order to find the highest level of biosurfactant production by *Kocuria rosea ABR6*, a two-stage experiment was designed. In the first stage, 40 tests were performed and after that 15 tests. Variable factors in these tests were pH, aeration speed and incubation. Based on these results, if the conditions are pH 9, aeration speed 120 rpm and incubation 72 h, the production of biosurfactant will be 100%.

### The effect of different factors in three dimensions

**Fig. 5:**
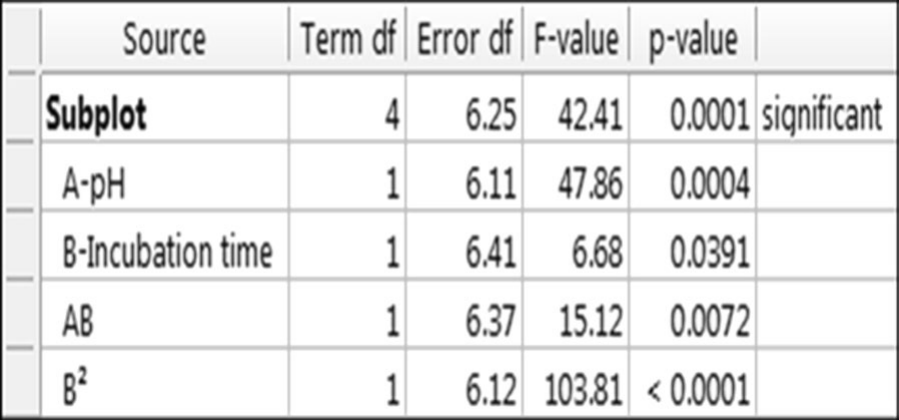
the analysis of variance ANOVA in biosurfactant production by *kocuria rosea* ABR6

**Fig. 6:**
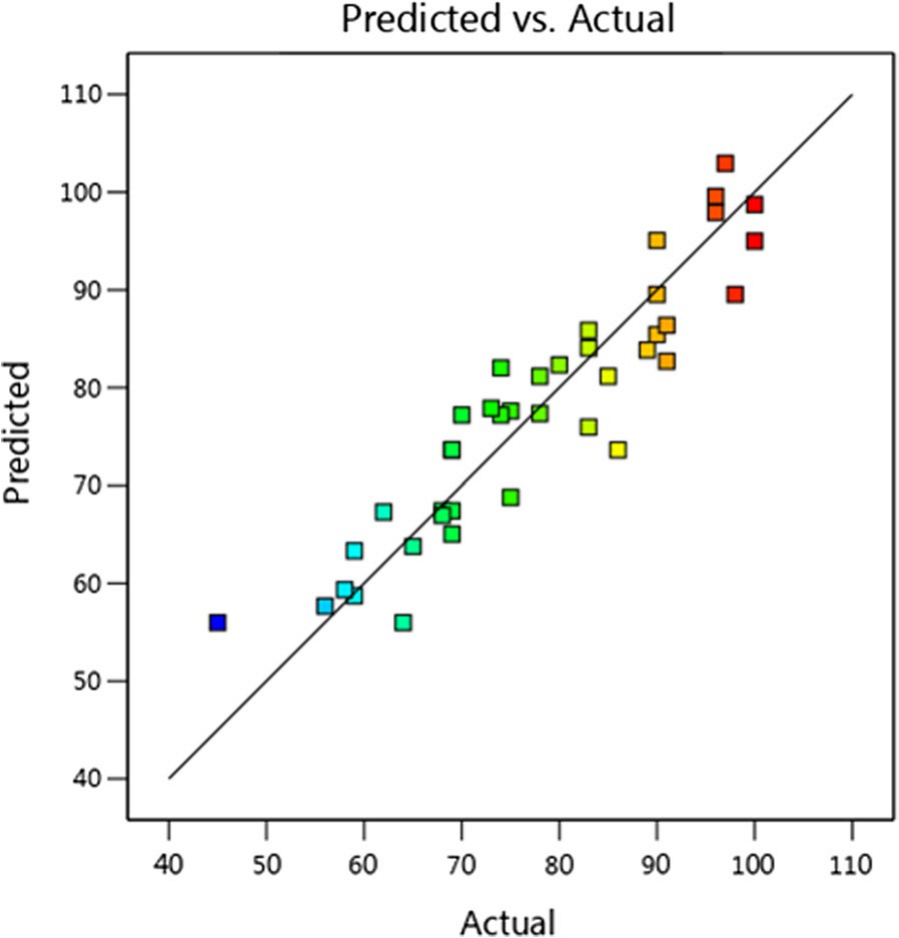
Actual and predicted values of biosurfactant production in RSM software by *Kocuria rosea* ABR6

**Fig. 7:**
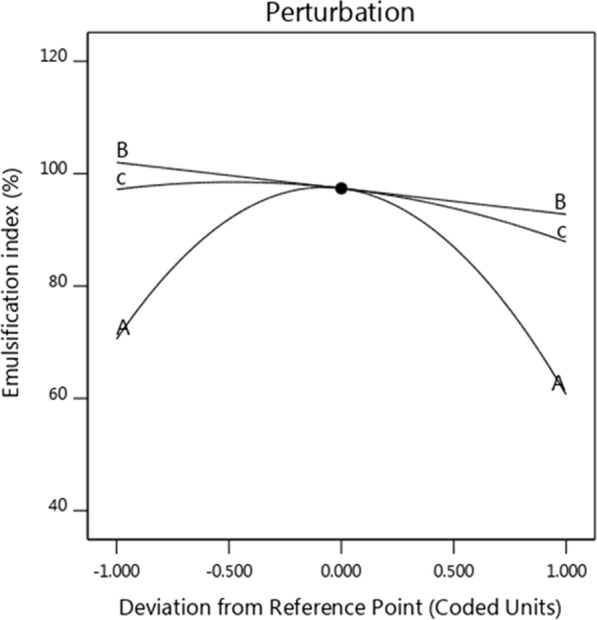
Impact of factors, A: pH, B: Incubation time, C: Agitation rate

Statistical analysis and analysis of variance (ANOVA) in the second stage of optimizing biosurfactant production by *Kocuria rosea* ABR6 was proceed. Figure 5 shows, the statistical analysis and analysis of variance (ANOVA) that for each variable, the corresponding correlation coefficient is P-value and F-value. Based on regression analysis and based on the evidences, it was found that pH and incubation time were positive factors and the P-value was less than 0.05. In general, the model was significant and significantly affected the production of biosurfactants. Figure 6 illustrated actual and predicted amounts of biosurfactant production found to be in linear form. Figure 7 shows, the effect of pH, agitation rate and incubation time factors on producing biosurfactant and emulsification index. As the slope of the invoice line increases, the effect of that factor on the response will be steeper, as well as, the Smoother line slope shows the effect of that factor on response was less. In this experiment, the representative factor, pH had a steep line slope and other factors like incubation time and agitation rate had slower line slope, therefore, they have less effect on the production of biosurfactants. Also, the production equation of biosurfactant based on the tested factors is as follows. According to this equation and the selection of other values, the amount of production can be predicted.

$$ {\text{Y }} = 93/71 - 71/0{\text{A}} + 26/{\text{3 B }} - 77/{\text{8C}} - 29/{\text{4AB}} + 48/{\text{7 A}}^{{2}} $$

Y = Biosurfactant production; A = pH; B = Incubation time; C = Agitation rate.

## Discussion

Nowadays, the importance of oil pollution is obvious. Oil, and especially oil sludge, is contamination sources in water and soil areas. For this purpose, a biological solution to remove oil sludge seems necessary and logical way. The diversity of bacteria are able to degrade oil and oil sludge, but obviously the bacteria that are native to the refinery they carry out this process better. Therefore, in this study, we isolated the native bacteria of the reservoir from storage tank and checked out the ability to produce biosurfactants, biodegradation and lubrication. In the present current study, a pipeline was designed in laboratory condition, biosurfactant that produced by *Kocuria rosea* ABR6 led to increasing movement of the crude oil in the pipelines. There are not many articles about effects of biosurfactant on crude oil lubrication by bacteria. In present study, we succeed to isolate 30 strains from tanks located in Isfahan Petroleum Refinery, by which 11 strains had the ability to produce biosurfactant. The Isolate named W11 was known as the best biosurfactant producer. The result of 16s rDNA shows, isolated W11 had been *Kocuria rosea*. The accession number of MK100469 confirmed by gene bank global and named *Kocuria rosea* ABR6. In order to identify species of bacteria, some routine biochemistry test was done, as well as, Jessim et al. (2020). the biochemistry test were helpful but they weren’t determiner, and because has also been observed that various species of *Kocuria* react differently to routine biochemical tests, so we had needed an advance molecular method and selected 16s rDNA (Kandi 2016). Advance molecular method amplicon 16s rDNA from isolated strain was sequenced, and the information was searched using NCBI-BLAST search tool to identify of the strain type. Based on results TLC and FTIR and the variety of materials present in the biosurfactant, its chemical nature was investigated and these results pointed to the lipopeptide nature. Dhasayan et al. (2015) demonstrated that in FTIR analysis, the biosurfactant obtained from B. amyloliquefaciens MB-101 was similar to the lipopeptide structures. Nature of biosurfactant demonstrate molecular characteristics and different applications, in this study, we introduce *Kocuria rosea* ABR6, as lubricant for crude oil, which reduced movement time of xrude oil. Ren et al. (2020) introduced biosurfactant SWPUEN-1 as a cleaning agent to treat oily sludge. During different experiments, we found there are the correlations among variable factors and decided to perform a statistical technique, named RSM to analyze them. RSM Analysis demonstrated, efficiency of biodegradation depends on the pH, agitation, aeration, carbon and nitrogen sources. The presence of heavy mental in oil sludge can be a hazardous and negative effect on the environment and human health (Hu et al. 2013). The potential of bacterial isolate in crude oil lubrication indicates the importance of biological usage *Kocuria rosea* ABR6 in petroleum industry. In this research, the designed pipeline applied to enhance the lubrication of crude oil through pipelines. It was explored this method employed to reduce viscosity and pressure drop to aid lubrication of crude oil pipeline, and it was successful. However, designing a pipeline to crude oil transport is depend on some factors e.g. the properties of the crude oil, distance dimension, cost, environmental problems and local and international regulations (Hart 2014). Many studies reported the isolation of biosurfactant-producing bacteria from sea sample, e.g. *Bacillus amyloliquefaciens SH20* and *Bacillus thuringiensis SH24* by Barakat et al. (2017), soil sample, *Acinetobacter junii B6* by Ohadi et al. (2017) and solar salt works, *Kocuria marina BS-15* by Sarafin et al. (2014). Due to the fact that the oil industry has a lot of bio-pollution, so the application of biological methods seems to be necessary. In the same study conducted by Karnwal (2017), *Kocuria rosea* was isolated and it can use as a pollution controlling factor in industrial and environmental applications. Sakshi et al. (2020) reported that *Kocuria Flava DTU-1Y* strain was isolated from contaminated soil and suggested it can improve the process in bioremediation problems.

The results of this study demonstrated *Kocuria rosea ABR6* as a native bacterium isolated from Esfahan Oil Refining Company is able to produce biosurfactant that is able to recover crude oil to 35 percentages and reduce the incidence of environmental pollution by crude oil. Producing biosurfactant in crude oil caused the acceleration movement in the pipeline from 64 to 39 s in the laboratory condition. Optimized conditions will be leading to significant increase in biosurfactant production. Therefore, the isolated bacterium in the current study is able to be a reasonable candidate to produce lipopeptide biosurfactant in industrial applications.

## Data Availability

All the information was archived in the additional files.
